# Immunological cross-reactivity between olive and grass pollen: implication of major and minor allergens

**DOI:** 10.1186/1939-4551-7-11

**Published:** 2014-05-08

**Authors:** Barbara Cases, Maria Dolores Ibañez, Jose Ignacio Tudela, Silvia Sanchez-Garcia, Pablo Rodriguez del Rio, Eva A Fernandez, Carmelo Escudero, Enrique Fernandez-Caldas

**Affiliations:** 1 Research and Development Department, Inmunotek s.l., Avda. Punto Mobi, 5., Alcalá de Henares (28871), Madrid, Spain; 2 Allergy department, Hospital Infantil Universitario Niño Jesús, Av de Menéndez Pelayo, 65., Madrid (28009), Spain

**Keywords:** Cross-reactivity, Minor allergens, *Olea europaea*, Panallergen, *Phleum pratense*, Pollen

## Abstract

**Background:**

Grasses and olive trees are the most common sources of allergenic pollen worldwide. Although they share some allergens, there are few studies analyzing the in vitro cross-reactivity between them. The aim was to define the cross-reactivity between *Olea europaea* and *Phleum pratense* using well-characterized sera of allergic children from Madrid, Spain.

**Methods:**

66 patients (mean age 10.32+/−4.07 years) were included in the study. All suffered from rhinoconjuntivitis and/or asthma and had a positive skin test and/or specific IgE determination to olive and grass pollen. Serum sIgE to individual allergens was conducted and sIgE against different grass species and olive was also determined by ELISA. Inhibition assays were performed using two serum sources, containing, or not, sIgE to minor allergens. Mass spectrometry analysis was performed in both extracts.

**Results:**

59/66 (89.39%) children had a positive sIgE determination by ELISA to grasses and 57/66 (86.36%) to olive pollen. There was no significant correlation between sIgE levels to grass and olive. Inhibition assays demonstrated no cross-reactivity between *P. pratense* and olive pollen when using the pool containing mainly sIgE to major allergens, whereas minimal to moderate cross-reactivity was detected when the serum contained high sIgE titers to minor allergens. Proteomic analyses revealed the presence of 42 common proteins in grasses and olive pollens.

**Conclusion:**

No in vitro cross-reactivity was observed when sIgE was mainly directed to major allergens. In our population, sensitization to olive and grasses is not due to cross-reactivity. The contribution of the major allergens seems to be determinant.

## Background

Allergy to grasses is the most important cause of pollinosis worldwide. Eleven allergen groups have been described in grasses [1]. Groups 1 (Subfamily of b-expansins) and 5 (heterogeneous proteins with ribonuclease activity) constitute the most immunodominant allergens with the highest prevalence of IgE binding and greatest sIgE binding capacity in children [2,3]. Groups 2, 3, 4 (berberine bridge enzymes) [4], 6, 7 (calcium binding proteins), 10 (cytochrome c), 11 (trypsin inhibitor, Ole e 1-related protein), 12 (profilins) and 13 (polygalacturonase) are also important allergens. However, Phl p 4, 7, 11, and 12 are not grass-specific.

Sensitization to olive pollen is also an important cause of pollinosis in Mediterranean countries and in the United States of America, especially in California [5]. Currently, 13 allergens have been described in *O. europaea;* 12 from pollen and one (thaumatin) as a food allergen from the olive fruit [6]. Ole e 1 is the major allergen*,* recognized by more than 70% of olive sensitized patients [7] and it has been proposed as a diagnostic marker for primary sensitization to *Oleaceae*[8]. Other allergens, such as profilin (Ole e 2), polcalcins (Ole e 3, Ole e 8), glucanases (Ole e 4, Ole e 9), superoxide dismutase (Ole e 5), lipid transfer proteins (Ole e 7), glycosyl hydrolases (Ole e 10), pectin methylesterase (Ole e 11), and Ole e 6, whose biological function is still unknown, have also been described.

Panallergens are responsible of a large number of cross-reactivity reactions among different species. Regarding a potential cross-reactivity between olive and grasses, the most likely implicated proteins are trypsin inhibitors, profilins and calcium binding proteins [9,10] corresponding to Ole e 1, Ole e 2, Ole e 3 and Ole e 8 in *O. europaea* and to groups 11, 12 and 7 in grasses. The scarce bibliography investigating cross-reactivity between grasses and *O. europaea*, suggests cross-reactivity between olive pollen and non-related species, including grass pollens [11,12].

Grasses and olive trees pollinate approximately at the same time in Spain. Therefore, to decide which one these pollens is producing clinical symptoms in polysensitized patients is complicated. However, we have previously demonstrated that olive and grass pollen mono-sensitized patients exhibit clinical symptoms during their respective, overlapping pollen seasons, suggesting the clinical relevance of these sensitizations [13,14].

The main objective of this study was to analyze cross-reactivity between olive and grass pollen extracts using well characterized sera of children in an area where both allergens are clinically relevant and endemic.

## Methods

### Patient population

Sixty six pediatric patients, 47 males (71.21%), with a mean age of 10.32 years ± 4.07 SD, consecutively evaluated in the outpatient clinic at the Niño Jesús Hospital, Madrid, Spain, were entered in the study. They were included if they fulfilled the following criteria: 1) less than 18 years, 2) a clinical history of allergic rhinoconjunctivitis and/or asthma during the grass and olive pollen season (May-June) of two years or more requiring treatment and 3) positive SPT and/or sIgE against grass and olive pollen. Oral and written consent was obtained from the parents, and by the patient if older than 12 years of age, to donate a blood sample to perform sIgE determinations related to their allergies. Exclusion criteria were previous treatments with immunotherapy with grass and/or olive extracts. Protocol number INM-RCA-2011-01 received IRB approval to be conducted.

### Skin tests and sIgE determination by ImmunoCap

Skin tests with a commercial battery of inhalant allergens including different grasses (*Phleum, Lolium* and *Cynodon*), olive pollen, palm tree profilin, dust mites, molds and animal dander (ALK-Abelló, Madrid, Spain) were performed to all patients using disposable 1 mm tip lancets. Results were read after 15 minutes, and a wheal diameter of 3 mm, or greater, was considered positive. Histamine (10 mg/mL) and saline SPT were used as positive and negative controls [15] (Table 1).

**Table 1:** Summary of the results of the sIgE determinations by SPT (A) and InmunoCap system (B)


**A**							
**SPT**		***P. pratense***		***O. europaea***		**Palm profilin**	
**Patients tested**		65 (98.48%)		65 (98.48%)		46 (69.69%)	
**Positive patients**		65 (100%)		63 (96.92%)		19 (41.30%)	
**B**							
**InmunoCAP**	***P. pratense***	**rPhl p 1**	**rPhl p 5**	**rPhl p 7**	**rPhl p 12**	***O. europaea***	**nOle e 1**
**Patients tested**	57 (86.36%)	55 (83.33%)	55 (83.33%)	43 (65.15%)	45 (68.18%)	55 (83.33%)	55 (83.33%)
**Positive patients**	57 (100%)	52 (95.55%)	32 (58.18%)	6 (13.95%)	14 (31.11%)	55 (100%)	52 (96.92%)

Results are expressed in percentage of positives.

Serum sIgE determinations to *Phleum Pratense*, *Olea europaea* and individual allergens (rPhl p 1, rPhl p 5, rPhl p 7, rPhl p 12 and nOle e 1) were performed by the CAP-System FEIA TM (Thermofisher Scientific, Spain). Results were considered positive when sIgE levels were above 0.35 kUA/l (Table 1). These analyses were part of the routine evaluation of the patients.

### Extracts for ELISA and ELISA Inhibition determinations

Protein extracts from *O. europaea* and *P. pratense* were obtained from acetone defatted pollens (IberPolen, Jaén, Spain). Briefly, extractions were performed 1:40 (w/v) in PBS buffer for 12 hours at 4°C under magnetic stirring conditions. After centrifugation at 10,000 rpm for 15 minutes, pellets were discarded and the supernatants sterile filtered through 0.22 μm cellulose acetate filters (Sartorius Stedim, Göttingen Germany). Extracts were dialyzed by ultrafiltration using a 5 kDa membrane (Pall, NY, USA) and freeze dried. The protein content was determined by the Bradford assay [16]. The protein concentration was 2.27 mg/ml for *O. europaea* and 5.4 mg/ml for *P. pratense.*

### ELISA and ELISA Inhibition

ELISA and ELISA inhibition assays were conducted in 96-well plates (Microlon, high binding, Greiner bio-one, Germany). Briefly, 1 μg of protein per well diluted in coating buffer (0.05 M carbonate bicarbonate buffer pH = 9.6) was used as solid phase. Plates were incubated overnight at 4°C, washed with PBS-tween 0.25% and blocked for 1 hour with PBS containing 0.25% Tween-20 and 2% BSA. Individual sera were added diluted 1/2 in blocking buffer and incubated for 2 hours at RT. Finally, plates were washed and incubated with a monoclonal mouse antihuman IgE peroxidase conjugated antibody (Southern Biotech, USA) diluted 1:2000 in blocking buffer. IgE reactivity was detected by the addition of OPD (Sigma-Aldrich, USA). Color development was stopped with HCl and plates were read at 495 nm in a Multiskan EX reader (Thermo Electron). Results were considered positive, when O.D. of a specific serum was 3 times higher than a negative control, which consisted of a serum pool of 7 non atopic patients.

ELISA inhibition assays were conducted in plates previously coated with 1 μg of protein per well of *P. pratense* or *O. europaea* extracts. Two types of sera were used in the ELISA Inhibition experiments. Serum pool 1 consisted of a mixture of all positive sera (including serum 11) and contained very low titers of sIgE to rPhl p 7 and rPhl p 12. It was used at a final dilution of 1/10. As a control of these inhibition experiments, we also performed inhibition experiments using another serum (serum 11) separately, which contained high sIgE titers to nOle e 1, to rPhl p 1 and rPhl p 5 and to rPhl p 7 and rPhl p 12 (see Additional file 1: Table S1). Both serum sources were previously titrated to assure the sensitivity and comparability of the assay. Serial 2-fold dilutions of both extracts ranging from 200 μg/ml to 0.01 μg of protein/ml were used as inhibitors.

### Mass spectrometry analysis

Mass spectrometry analyses were carried out in the proteomic facilities from the Parapléjicos Hospital (Toledo, Spain), with a previously described method. Briefly, protein samples were diluted in 8 M urea. After reduction and alkylation, proteins were trypsin digested. The peptides were separated on nano-LC system and collected fractions were collected and spotted on a blank MALDI sample plate. MS and MS/MS analysis of offline spotted peptide samples were performed using the Applied Biosystems 4800 plus MALDI TOF/TOF Analyzer mass spectrometer. Peptide and protein identifications were performed using ProteinPilotTM Software V 2.0.1 (Applied Biosystems) and the Paragon algorithm [17]. MS/MS spectrums were searched against different databases (SwissProt and Uniprot). The confidence percentage calculated by the software (unused score) reflects the probability of “false positives”, meaning that at the 90% confidence level, there is a false positive identification probability of about 10%.

### Statistical analysis

The Spearman correlation coefficient was determined (GrahPad Prism 5.03 Software) to analyze the correlation coefficients between *P. pratense* and olive extracts used in the study.

## Results

### Skin tests and ImmunoCAP sIgE determination

Skin tests to *P. pratense* were performed in 65 patients and were positive in all of them. Skin tests to *O. europaea* were also performed in 65 patients and 63 were positive (96.92%). Palm tree profilin was tested in 46 patients and 41.30% of them had a positive SPT result (Table 1).

**Figure 1:**
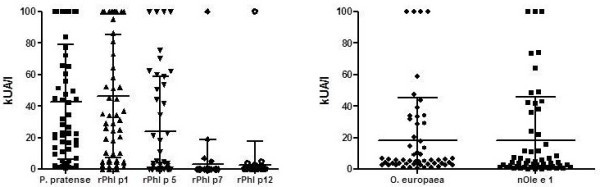
**Serum sIgE levels to *P. pratense *** **(n = 57), *O. europaea *** **(n = 55) and purified allergens measured by the ImmunoCAP system.**

Serum sIgE to *P. pratense* was positive in all tested patients (n = 57); rPhl p 1 (n = 55) and rPhl p 5 (n = 57) sIgE were positive in 95.55% and 58.18%, respectively; rPhl p 7 (n = 43) was positive in 13.95% and rPhl p 12 (n = 45) in 31.11%. Serum sIgE determinations to *O. europaea* were positive in all patients and nOle e 1 (n = 55) in 94.54% of sera tested (Figure 1).

### ELISA

**Figure 2:**
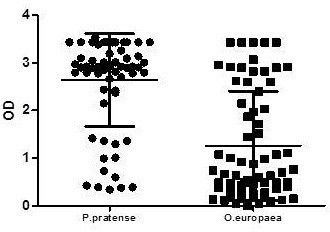
**Serum sIgE binding to *P. pratense *** **and *O. europaea *** **by ELISA in individual sera.**

Serum sIgE to *P. pratense* and *O. europaea* was measured using ELISA. A positive sIgE determination was observed in 89.39% (59/66) and 86.36% (557/66) children, respectively. The mean and standard deviation of sIgE levels (OD) were 2.64 ± 0.97 for *P. pratense* and 1.26 ± 1.14 for *O. europaea* (Figure 2).

### Cross reactivity assessment

**Figure 3:**
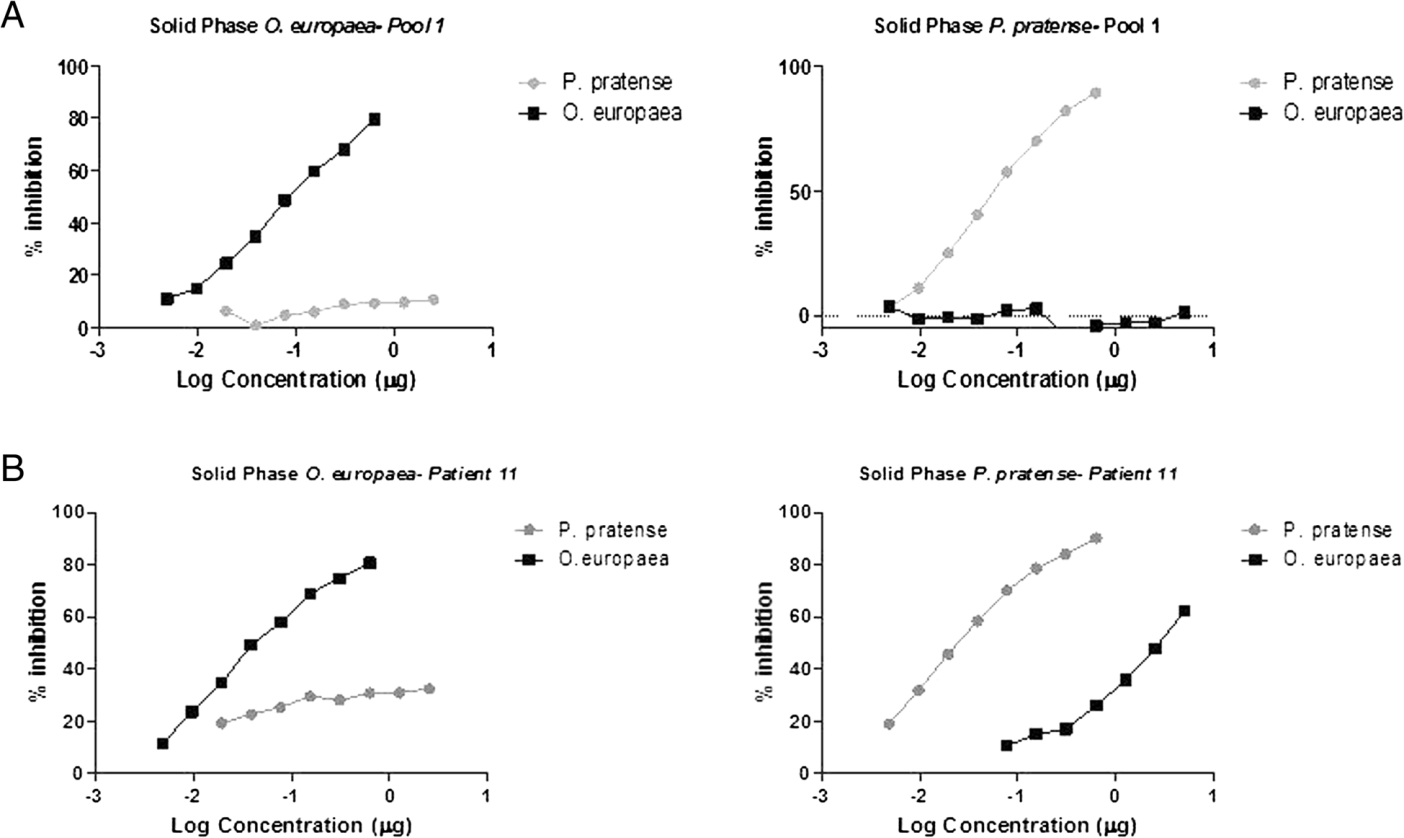
A) Inhibition ELISAs using a serum pool (pool 1) and an individual serum (patient 11) sensitized to panallergens (B).

Inhibition assays carried out with *O. europaea* and *P. pratense* did not show any inhibition when serum pool 1 was used (Figure 3A). However, when serum 11 was used a moderate degree of cross-reactivity was observed between both extracts (Figure 3B). *O. europaea* inhibited up to 61% the sIgE binding to *P. pratense* and *P. pratense* inhibited up to 33% sIgE binding to *O. europaea*.

### Mass spectrometry

Results revealed the presence of common proteins in *O. europaea* and *P. pratense* extracts. Among the peptides identified, several belonged to allergenic proteins. MS/MS analysis also confirmed the existence of at least 42 common proteins in both extracts and the presence of numerous potential allergenic molecules, which have been described in other allergen sources (Table 2).

**Table 2:** **Common proteins identified in *O. europaea *** **and *P. pratense *** **extracts by mass spectrometry (n = 42)**

**Common proteins identified in *O. europaea *** **and *P. pratense *** **pollen extracts**	
-3-3 protein	Ketol-acid reductoisomerase
5-methyltetrahydropteroyltriglutamate--homocysteine methyltransferase, putative	L-ascorbate peroxidase 2, cytosolic
70 kDa heat shock cognate protein 2	Luminal binding protein
Heat shock 70 kDa protein	Malate dehydrogenase, cytoplasmic (Fragment)
Alpha-1,4-glucan-protein synthase [UDP-forming] 2	Methionine synthase
Ascorbate peroxidase 2	Monodehydroascorbate reductase
AT1G56340 protein	mRNA, clone: RTFL01-12-H18
ATP citrate lyase	mRNA, clone: RTFL01-39-D20
ATP-citrate synthase, putative	Nucleoside diphosphate kinase
Auxin-induced protein PCNT115	Os01g0300200 protein
Beta-D-glucosidase	Pectinesterase
Calcium binding protein- Calreticulin	
cDNA clone:001-017-G06, full insert sequence	Phopholipase D (Fragment)
DnaK-type molecular chaperone hsp70-rice	Phosphoglycerate kinase (Fragment)
Elongation factor (Fragment)	Polygalacturonase (Fragment)
Enolase	Profilin
Exo-1,3-beta-glucanase	Soluble inorganic pyrophosphatase
Fructokinase-like protein (Fragment)	Triosephosphate isomerase
	Trypsin inhibitor
Fructose-bisphosphate aldolase (Fragment)	UDP-glucose pyrophosphorylase
Inorganic pyrophosphatase (Fragment)	Uridylate kinase plant, putative
Isocitrate dehydrogenase [NADP]	UTP-glucose-1-phosphate uridylyltransferase

### Statistical analysis

No significant correlation was observed between olive and *P. pratense* sensitization (r = 0.106; p = 0.4).

## Discussion

In this paper we analyzed the *in vitro* cross-reactivity between *P. pratense* and *O. europaea* using sera from allergic children. The population used in our study had 4 main characteristics. First, they were all children, which could have affected the overall sensitization pattern; second, they all lived in a region where both allergen sources are clinically relevant; third, 94.5% of the patients were sensitized to Ole e 1, which is a marker of sensitization to Olive [8] and fourth, the vast majority of the patients (98.1%, of the patients tested) were also sensitized to grass groups 1 and/or 5, of which 94.5% were sensitized to Phl p 1 and 58.2% to Phl p 5. These data are in agreement with other authors, which have shown a greater prevalence of sensitization to group 1 than to group 5, in both pediatric and adult patients [18].

The study by Sekerkova *et al*. was performed in a population of grass sensitized children and adults. The authors demonstrated no significant differences in the rate of sensitization to Phl p 1 in adults and in children (90.2% and 93.8%, respectively). However, when comparing patients regarding the prevalence of specific IgE antibodies against Phl p 5, the authors described a 20% higher incidence of specific antibodies to this allergen in the group of adults (79.1%) *versus* children (59.8%). This incidence found in children by Sekerkova *et al.* is in accordance to the results obtained in our study (Table 1).

Strikingly the prevalence of sIgE to the minor allergens Phl p 7 and Phl p 12 was also very similar in both studies. Molecular diagnosis supports the hypothesis that panallergens such as profilins and polcalcins can be considered as minor allergens in both, olive and grasses, with a minority of patients sensitized, in variable degrees to those allergens (13.95% to polcalcin and 31.11% to profilin). Other approaches that support no cross-reactivity are the statistical analyses, which show no correlation between sensitization to olive and the grass species, in spite of the large number of common proteins as shown by mass spectrometry results.

We used two serum sources from the same patient population, who resided in an area where both allergen sources are important to assess cross-reactivity. Serum pool 1 contained high levels of sIgE to the major allergens Phl p 1 and Phl p 5 and to Ole e 1 and low levels to Phl p 7 and Phl p 12. Serum sIgE to olive profilins and polcalcins could not be analyzed due to a lack of commercially available reagents. On the other hand, serum 11 contained high sIgE titers to both major allergens in *P. pratense* and *O. europaea* and additionally to Phl p 7 and Phl p 12 was employed as an individual control and. This serum was the only one with these characteristics in this patient population, which may represent a minority of patients. We acknowledge that the use of only one serum to compare with a serum pool may not be adequate. However, we believe that serum 11 is representative of a group of patients that are highly sensitized to minor and major allergens. Further studies should evaluate the individual contribution of profilin and/or polcalcins to the overall clinical sensitivity of these patients and to the allergenicity of the extracts.

Cross-reactivity studies confirmed 2 different inhibition patterns. It is suggested that if patients are not sensitized to minor allergens, allergen cross-reactivity is exclusively mediated by major allergens and, therefore, no cross-reactivity is seen. However, when using a serum with high sIgE titers to panallergens, the overall cross-allergenicity of the allergen extracts varies, due to the contribution and recognition of these minor allergens. Although we have only shown this phenomenon between Olive and *P. pratense* pollen extracts, these findings may be extrapolated to other plant allergen sources, including fruits, in which the presence of these minor allergens has been demonstrated.

In order to assure that both allergen extracts used in the cross-reactivity studies were representative, we conducted mass spectrometry analysis. As a result of these analyses, we confirmed the presence of Phl p 1, Phl p 2, Phl p 3, Phl p 4, Phl p 5, Phl p 6, Phl p 7, Phl p 11, Phl p 12, and Phl p 13 in the *P. pratense* pollen extract. In the case of olive pollen, proteomic analysis allowed the identification of Ole e 1, Ole e 2, Ole e 4, Ole e 5, Ole e 6, Ole e 9, Ole e 10 and Ole e 11. Surprisingly, Ole e 8 and Ole e 3 were not identified. This may be due to the low concentrations of these proteins in the olive pollen extracts. Except for Ole e 1, which could represent approximately 20% of the total protein content in the pollen, other allergens are present in reduced quantities. This may be the case of Ole e 8, which constitutes less than 0.05% of the total protein in the extract [19]. Another important issue to consider is the location of the allergen within the pollen grain. It has been shown that Phl p 1, 4, 5, 6, and 12, were detected in extracts of pollen and pollen cytoplasmic granules (PCGs), whereas Phl p 11 was found only in PCGs, and Phl p 2 as well as Phl p 13 only in pollen extracts [20].

Table 2 summarizes a list of common proteins detected by mass spectrometry in both extracts. This list contains only those proteins with a confidence percentage of 90%. Non allergenic and potentially allergenic proteins were identified. Potential allergens, which have been described in other plant and/or non-vegetable sources, could be responsible of allergenic cross-reactivity between different species. For instance, triose-phosphate isomerase, malate hydrogenase and profilins were described as major allergens in watermelon [21,22]; Triose phosphate isomerase was also described in german cockroach (*Blattella germanica*) [23]; Enolase, in latex [24], *Candida albicans*[25]*Sacharomices cerevisiae*[26], *Aspergillus fumigatus*[27], *Alternaria alternata Cladosporium herbarum*[28] and *Blattella germanica*[23]; Polygalacturonase, in *cuppresaceae*[29], tomato [30], and *Platanus acerifolia*[31] and metylesterase in *Salsola kali*[32], among others. Interestingly, 1,3-beta-glucanases were identified in both allergen extracts. Ole e 9 and Ole e 4 are 1,3-beta-glucanase in Olive pollen. However, glucanases have not been described as allergens in grass pollen extracts. Ole e 1-related proteins were also identified in *P. pratense* and olive pollen extracts. However, while Ole e 1 is a major allergen in Olive pollen, Phl p 11, is a minor allergen in grasses.

## Conclusions

Taken into consideration the results of this study, we can conclude that there is no *in vitro* cross-reactivity between *O. europaea* and *P. pratense* pollen extracts when using serum of children mainly sensitized to major allergens and low levels of sIgE to minor allergens. This kind of patients represents the majority of the grass sensitized population in Spain. However, when the serum of a patient highly sensitized to minor allergens was used, some cross-reactivity could be detected. This fact may have clinical implications in the selection of extracts for immunotherapy, and therefore, component resolved analysis would be of great help to identify patients sensitized to minor allergens. To a large extent, sensitization to olive and grasses is not due to cross-reactivity but is a consequence of co-sensitization. A minority of patients recognize common allergens in both allergen sources in variable degrees.

## Competing interests

The author declares that they have no competing interests.

## Authors’ contributions

BC contributed to the conception and design of the study, data generation, analysis of the data, preparation and revision of the manuscript. MDI contributed to the conception and design of the study data generation and critical revision of the manuscript. JIT contributed data generation and analysis of the data and critical revision of the manuscript. SSG contributed to the conception and design of the study data generation and critical revision of the manuscript. PRR contributed to the conception and design of the study data generation and critical revision of the manuscript. EAF contributed data generation, analysis of the data and critical revision of the manuscript. CE contributed to the conception and design of the study data generation and critical revision of the manuscript and EFC contributed to the conception and design of the study, data generation, analysis of the data and preparation and critical revision of the manuscript. All authors had read and approved the final version of the manuscript.

## Supplementary Material

Additional file 1: Table S1 Clinical data of the patients. ImmunoCAP units are expressed in kU/L. Click here for file
