# Supplementary material for: Immunological cross-reactivity between olive and grass pollen: implication of major and minor allergens
Source: World Allergy Organ J. 2014 May 8;7(1):11. doi: 10.1186/1939-4551-7-11 (PMC4045862; doi:10.1186/1939-4551-7-11)
Supplement: Additional file 1: Table S1 — Clinical data of the patients. ImmunoCAP units are expressed in kU/L. [file 1939-4551-7-11-S1.docx]

Additional file 1 Table S1: Clinical data of the patients. ImmunoCAP units are expressed in kU/L.

| **Patient** | **Symptoms** | **Prick Phleum** | **Prick Olea** | **Prick Profilin** | **CAP Phleum** | **CAP rPhl p1** | **CAP rPhl p 5** | **CAP rPhl p7** | **CAPç rPhl p12** | **CAP Olea** | **CAP**  **nOle e1** |
| --- | --- | --- | --- | --- | --- | --- | --- | --- | --- | --- | --- |
| **1** | **R, A** | P | P | N | 55,6 | 100 | 0 | 0 | 0,49 | 100 | 100 |
| **2** | **R, A** | P | P | N | 11 | 10,2 | 0 | 0 | 0 | 4,54 | 1,64 |
| **3** | **R, A** | P | P | ND | 100 | ND | ND | ND | ND | ND | ND |
| **4** | **R, A** | P | P | ND | 0,63 | 0 | 0 | 0 | 0 | 3,56 | 1,94 |
| **5** | **R, A** | P | P | ND | ND | ND | ND | ND | ND | ND | ND |
| **6** | **R, A** | P | P | ND | ND | ND | ND | ND | ND | ND | ND |
| **7** | **R, A** | P | P | N | 1,95 | 2,49 | 0 | 0 | 0 | 0,35 | 0 |
| **8** | **R, A** | P | P | N | 100 | 100 | 2,17 | 0 | 0,35 | 59 | 64,1 |
| **9** | **R** | P | P | N | 100 | 100 | 100 | 0,35 | 0 | 9,34 | 7,36 |
| **10** | **R, A** | P | P | N | 65,4 | 86,4 | 44,2 | 0 | 0 | 6,07 | 8,62 |
| **11** | **R, A** | P | P | P | 100 | 100 | 100 | 100 | 100 | 100 | 73,8 |
| **12** | **R** | P | N | ND | 100 | 34,1 | 100 | 0 | 0 | 6,7 | 0,94 |
| **13** | **R** | P | P | P | 5,49 | 1,47 | 0 | 0 | 4,51 | 29,1 | 36 |
| **14** | **R, A** | P | P | N | 77,4 | 100 | 0 | 0 | 0 | 32 | 48,6 |
| **15** | **R** | P | N | N | 100 | 100 | 70,2 | 0 | 0 | 2,9 | 0,42 |
| **16** | **R** | P | P | N | 21,3 | 18,1 | 3,91 | 0 | 0 | 47,7 | 73,5 |
| **17** | **R, A** | P | P | N | 100 | 100 | 100 | 0 | 0 | 39,1 | 42,2 |
| **18** | **A** | P | P | N | 100 | 95,4 | 100 | 18,9 | 0 | 17,9 | 0,47 |
| **19** | **R** | P | P | P | 100 | 100 | 60,1 | 6,85 | 0,5 | 44 | 41,7 |
| **20** | **R** | P | P | N | 19,7 | 52,4 | 0,35 | ND | ND | 2,89 | 3,02 |
| **21** | **R, A** | P | P | P | 23,1 | 5,43 | 18,4 | ND | ND | 3,92 | 1,17 |
| **22** | **R, A** | P | P | N | 100 | 100 | 5,29 | ND | ND | 6,28 | 4,88 |
| **23** | **R** | P | P | ND | ND | ND | ND | ND | ND | ND | ND |
| **24** | **R** | P | P | N | 100 | 100 | 50,8 | 0 | 0 | 5,29 | 1,84 |
| **25** | **R, A** | P | P | P | 28,4 | 34,4 | 2,97 | 0,35 | 1,02 | 1,04 | 0,47 |
| **26** | **R, A** | P | P | P | 84,1 | 73,2 | 53,6 | ND | ND | 6,73 | 0,92 |
| **27** | **R** | P | P | N | 0,86 | 2,12 | 0 | 0 | 0 | 3,65 | 3,89 |
| **28** | **R, A** | P | P | N | 34,4 | 31,1 | 21,3 | 0 | 0 | 3,18 | 2,6 |
| **29** | **R, A** | P | P | N | ND | ND | ND | ND | ND | ND | ND |
| **30** | **R** | P | P | ND | ND | ND | ND | ND | ND | ND | ND |
| **31** | **R** | P | P | ND | 16,6 | 45,5 | 0 | 0 | 0 | 1,29 | 1,23 |
| **32** | **R, A** | P | P | P | 22,2 | 29,3 | 0 | ND | ND | 4,5 | 0 |
| **33** | **R** | P | P | P | 1,84 | 0 | 4,9 | 0 | 0 | 5,78 | 4,08 |
| **34** | **R, A** | P | P | P | 3,68 | 4,9 | 0 | 0 | 0 | 3,82 | 2,78 |
| **35** | **R** | P | P | ND | ND | ND | ND | ND | ND | ND | ND |
| **36** | **R, A** | P | P | P | 26,9 | 51,3 | 0 | ND | ND | 13,8 | 15,6 |
| **37** | **R** | P | P | N | 51,4 | 24,4 | 34,7 | 0 | 0 | 10,5 | 11,1 |
| **38** | **R, A** | P | P | P | 65,5 | 26,1 | 62,5 | 0 | 0 | 5,44 | 3,84 |
| **39** | **ND** | P | P | N | 17,4 | 58,4 | 0 | 0 | 0 | 34,2 | 43 |
| **40** | **R** | P | P | ND | ND | ND | ND | ND | ND | ND | ND |
| **41** | **R** | P | P | N | 1,02 | 4,68 | 0 | 0 | 0 | 100 | 100 |
| **42** | **R, A** | P | P | P | 13,7 | 29,3 | 0 | 0 | 0 | 28,7 | 24 |
| **43** | **R** | P | P | N | 5,19 | 5,7 | 0,35 | 5,06 | 0 | 14,8 | 11,5 |
| **44** | **R, A** | P | P | N | 31,9 | 64,6 | 0,35 | 0 | 0 | 2,72 | 2,72 |
| **45** |  | P | P | ND | 9,29 | ND | ND | ND | 0,35 | ND | ND |
| **46** | **R, A** | P | P | ND | 44,1 | 36,9 | 58,7 | 0 | 0,61 | 6,57 | 6,67 |
| **47** | **R, A** | P | P | N | 100 | 100 | 75,6 | 0 | 1,67 | 100 | 100 |
| **48** | **R, A** | P | P | ND | 47,3 | 100 | 0 | 0 | 0 | 5,6 | 6,74 |
| **49** | **R, A** | P | P | P | 43,9 | 81,4 | 0 | 0 | 0 | 6,01 | 6,08 |
| **50** | **R, A** | P | P | P | 49,4 | 44,5 | 41,9 | 0 | 2,65 | 5,47 | 0,35 |
| **51** | **R, A** | P | P | N | 44,7 | 100 | 3,6 | 0 | 0 | 2,88 | 3 |
| **52** | **R, A** | P | P | P | 1,98 | 0 | 0 | 0 | 0,49 | 4,44 | 5,16 |
| **53** | **A** | P | P | P | 22,7 | 4,06 | 22,8 | 0 | 1,31 | 2,42 | 0 |
| **54** |  | P | P | ND | ND | ND | ND | ND | ND | ND | ND |
| **55** | **R** | ND | ND | ND | 6,98 | 10 | 0 | 0 | 0 | 9,17 | 11,5 |
| **56** | **R, A** | P | P | N | 2,1 | 4,73 | 0 | 0 | 0 | 3,07 | 4 |
| **57** |  | P | P | P | 1,05 | 1,67 | 0 | ND | ND | 1,3 | 1,36 |
| **58** | **R, A** | P | P | ND | 23,2 | 25 | 2,35 | ND | ND | 2,79 | 0,45 |
| **59** | **R, A** | P | P | P | 42,4 | 4,82 | 0 | 0 | 3,54 | 3,48 | 3,54 |
| **60** |  | P | P | N | 18,5 | 10,6 | 11 | 0 | 0 | 1,81 | 1,1 |
| **61** | **R, A** | P | P | ND | 12,8 | 19,4 | 3,48 | ND | ND | 1,21 | 0,92 |
| **62** | **R, A** | P | P | N | 60,6 | 52,3 | 62,2 | ND | ND | 4,02 | 2,24 |
| **63** | **R, A** | P | P | ND | 22,2 | 20,6 | 0 | ND | 0 | 20,4 | 22,1 |
| **64** |  | P | P | ND | 15,1 | 35,2 | 0 | ND | ND | 33,9 | 49 |
| **65** | **R, A** | P | P | P | 72,2 | 100 | 100 | 0 | 2,16 | 33,5 | 38,3 |
| **66** | **A** | P | P | ND | ND | ND | ND | ND | ND | ND | ND |

R: Rhinitis, A: Asthma, P: Positive, N: Negative, ND: Not Determined.
*P. pratense* major allergens: Phl p 1, Phl p 5; Phl p 7 (Polcalcin); Phl p 12 (Profilin).
